# Requirements for a Dashboard to Support Quality Improvement Teams in Pain Management

**DOI:** 10.3389/fdata.2021.654914

**Published:** 2021-05-14

**Authors:** Jeremy Opie, Maura Bellio, Rachel Williams, Maya Sussman, Petra Voegele, John Welch, Ann Blandford

**Affiliations:** ^1^Wellcome/EPSRC Centre for Interventional and Surgical Sciences (WEISS), University College London (UCL), London, United Kingdom; ^2^University College London Interaction Centre (UCLIC), London, United Kingdom; ^3^Critical Care Unit, University College London Hospital (UCLH), London, United Kingdom

**Keywords:** pain management, dashboards, critical care unit, electronic health record, quality improvement team

## Abstract

Pain management is often considered lower priority than many other aspects of health management in hospitals. However, there is potential for Quality Improvement (QI) teams to improve pain management by visualising and exploring pain data sets. Although dashboards are already used by QI teams in hospitals, there is limited evidence of teams accessing visualisations to support their decision making. This study aims to identify the needs of the QI team in a UK Critical Care Unit (CCU) and develop dashboards that visualise longitudinal data on the efficacy of patient pain management to assist the team in making informed decisions to improve pain management within the CCU. This research is based on an analysis of transcripts of interviews with healthcare professionals with a variety of roles in the CCU and their evaluation of probes. We identified two key uses of pain data: direct patient care (focusing on individual patient data) and QI (aggregating data across the CCU and over time); in this paper, we focus on the QI role. We have identified how CCU staff currently interpret information and determine what supplementary information can better inform their decision making and support sensemaking. From these, a set of data visualisations has been proposed, for integration with the hospital electronic health record. These visualisations are being iteratively refined in collaboration with CCU staff and technical staff responsible for maintaining the electronic health record. The paper presents user requirements for QI in pain management and a set of visualisations, including the design rationale behind the various methods proposed for visualising and exploring pain data using dashboards.

## 1. Introduction

One of the most difficult tasks associated with big data is that of organising, analysing, and presenting data to users in a manner that supports sensemaking (Venkatraman and Venkatraman, 2019). Visualisations are a means by which sensemaking can be achieved more rapidly than through text (Ware, 2004). Through the use of visualisation tools, data can be abstracted into meaningful visual representations (Blandford et al., 2014) that enable “ah HA!” moments to occur (Spence, 2007). Conceptual structures of the domain in which the users work promotes sensemaking, as understanding the way in which users think about their activities makes it possible to develop visualisations that capture this structure (Blandford et al., 2014).

One of the main stressors experienced by critically ill patients has been identified as pain, which has been attributed to poor pain management (Devlin et al., 2018). When patients are in severe pain, additional comorbidities can occur including confusion, delirium, compromised respiratory and cardiac function, and sleeplessness (Devlin et al., 2018). Conversely, if pain is over treated then compromised respiratory and cardiac function can still occur, as well as reduced consciousness and depression (Dahan and Teppema, 2003). It is therefore important that the correct dosage is given at the right time so as not to compromise patients' respiratory and cardiac functions. Evaluating and exploring pain data within the critical care unit can help to identify processes and actions to alleviate these issues.

A multidisciplinary team of clinicians in a London teaching Hospital set up a Quality Improvement (QI) project to assess pain management in the Critical Care Unit (CCU). Routinely collected data from Electronic Health Records (EHRs) was used to generate probes to inform unit practise, with the ultimate aim of creating a bespoke dashboard. The aim of this study was to understand the QI team's conceptual structures relating to pain management to inform the design of dashboards that support sensemaking. Twelve CCU healthcare professionals were interviewed and the transcripts of those interviews were analysed to identify how they currently interpret information and determine what information is important to them in decision making.

In section 2, we present related work specific to dashboards for QI teams; section 3 details the methods used in this study; section 4 presents the results specific to pain management for QI teams; section 5 presents proposed dashboard visualisations and outlines the design rationale behind each visualisation; in section 6, we discuss the implications and limitations of this work, with section 7 providing concluding remarks.

## 2. Related Work

Within the business sector, dashboards were developed to visualise important information to assist with decision making (Pauwels et al., 2009). Dashboards have also begun to be adopted in other fields, including healthcare. Healthcare dashboards are a type of health information technology (HIT) (Dowding et al., 2015) that present information relevant to the quality of patient care in a timely manner (Daley et al., 2013). Dashboards allow raw tabulated data to be organised into clear visuals that provide important information relevant to the user (Wexler et al., 2017). Within healthcare there are two main types of dashboards: *clinical dashboards* that focus on the performance of individual clinicians or patients (Dowding et al., 2018) and *quality dashboards* for evaluating the performance of a unit such as a ward (Kroch et al., 2006; Keen et al., 2018).

A number of *clinical dashboards* have been developed to provide specific information for clinicians on the status of their patients, including blood pressure (Stinson et al., 2012), lab results (Batley et al., 2011), diabetes (Koopman et al., 2011), acute respiratory infections (Linder et al., 2010), and radiography data (Morgan et al., 2008). Although quality dashboards are already used within hospitals (Weggelaar-Jansen et al., 2018), few have been discussed in the academic literature; probably the most notable example is QualDash (Elshehaly et al., 2020).

QualDash supports clinicians and managers by allowing them to explore data for QI from the Myocardial Ischaemia National Audit Project (MINAP) and the Paediatric Intensive Care Audit Network (PICANet), which are national audits. Data is accessed via an interactive web-based dashboard displaying key metrics on QualCards that visualise data using bar graphs, pie graphs, etc. QualDash was developed through interviews and co-design workshops with a number of clinicians and managers to produce seven design requirements: it must support pre-configured reusable queries for dynamic QualCard generation; each QualCard must have two states, entry-point and expanded; it needs to support GUI-based adaptability of subsidiary view measures; it must deliver data on time; incorporate data quality to identify missing or invalid data; support export of visualisations; and provide data privacy.

When designing *quality dashboards* Randell et al. (2020) provide five themes that should be included: choosing performance indicators, assessing performance, identifying causes, communicating from ward to board, and data quality. Each of these has specific requirements attached to it.

Choosing performance indicators allows users to decide which parameters are important to their inquiry. According to Randell et al., there are five requirements for assessing performance: use evidence-based standards if they exist; support identification and evaluation of trends over time; provide means to adjust the time period; support comparison against the national average; and provide means to select particular organisations when performing comparisons. For the theme of identifying causes, there are three requirements: allow users to drill down into data; provide access to additional information from other clinical areas; and support simultaneous interaction. Communicating from ward to board requires that outliers found in audits are easily identifiable at a corporate level, and data quality requires that data is provided in a timely manner and that staff trust the source of the data.

Roos-Blom et al. (2017) developed a web-based dashboard to improve the performance of pain management within the ICU. Staff would input feedback of barriers they faced and the software would produce suggestions and in some cases supporting documentation to help overcome the barriers. It was reported that, using this system, adequate pain management rose by 10% (Roos-Blom et al., 2019).

In summary, dashboards are being developed for healthcare, including the work carried out by Roos-Blom et al. on removing barriers to bedside pain management (Roos-Blom et al., 2017, 2019). However, to our knowledge, no prior work has developed a quality improvement dashboard to assist with understanding issues relating to pain management within a CCU.

This study seeks to understand the needs of quality improvement team members to develop dashboard proposals that can improve conceptualisation of data to better support sensemaking.

## 3. Methods

**Figure 1:**
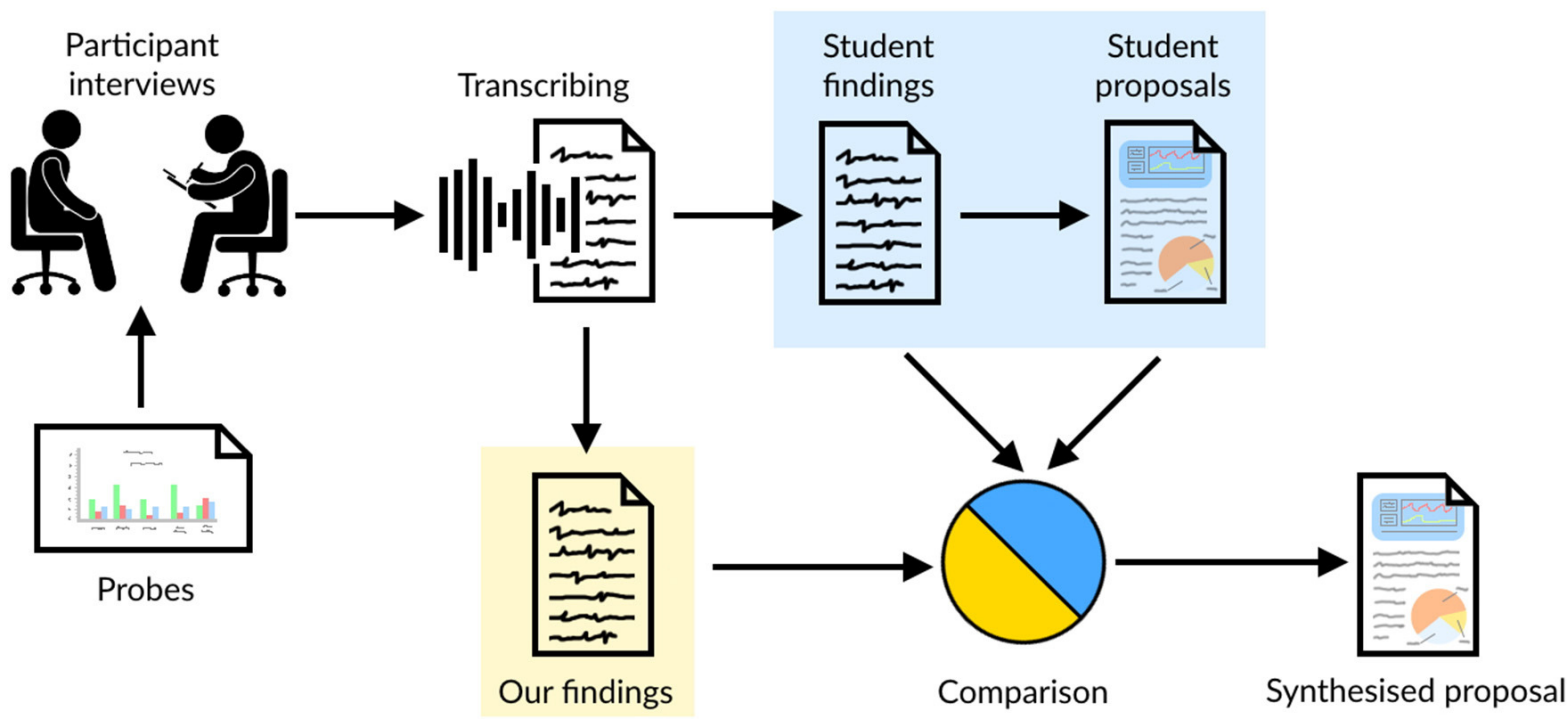
Visual representation of method used in this paper.

The aim of this research was to gain insights from CCU staff on how they manage patients' pain levels, and their understanding of probes, which consist of proposed dashboard infographics developed by the QI team. The research presented in this paper is based on interviews with healthcare professionals working in the Critical Care Unit (CCU), with a primary focus on pain management. Figure 1 depicts the method that was used during this study, highlighting: the interviews with healthcare professionals, which were conducted by Masters students; probes used during the interviews; the transcription of the interviews; and the students' findings and proposals they produced as part of their course-work. This research is a re-analysis of the interview transcripts with healthcare professionals. It was conducted independently of the students' results, before triangulating the findings by comparing the students' outcomes with our own, and reviewing the students' design proposals. This led to a synthesised design proposal built from the work of both the students and our independent analysis. Ethical clearance was obtained for the study carried out (UCLIC/1617/004/Staff Blandford HFDH), and permission for the secondary analysis and use of student data was obtained from all students.

### 3.1. Participants

Healthcare professionals were recruited based on their availability and willingness to participate in the study. They were also selected based on their role within the CCU to obtain a cross-section of the staff. Each of the participants is presented in Table 1; this includes their participation identification tag (PID), used to identify individual participants in this paper.

**Table 1:** List of participants and their roles in the hospital.

**PID**	**Participant profession**
P1	Physiotherapist
P2	Research Nurse
P3	Registered Nurse (Pain Management)
P4	Senior Nurse in Post-Anaesthesia Care Unit (PACU)
P5	Doctor in CCU
P6	Clinical Practice Facilitator
P7	Deputy Sister
P8	Honorary Consultant Nurse
P9	Junior Staff Nurse (Band 5)
P10	Practitioner Nurse (Band 7)
P11	Senior Nurse
P12	Senior Assistant in the CCU

### 3.2. Interviews

The interviews were semi-structured in a manner that prompted CCU staff to provide details focusing on the following: How they identify patients' pain? What systems are in place currently to assist them? What information do they use to help make decisions around pain management? How do they interpret the information in the probes? What information would be helpful to them to improve the probes? Twelve interviews were conducted with different healthcare professionals, each interview being conducted by a separate group of students (2–3 students per group). Prior to conducting the interviews, students prepared questions for healthcare professionals that focused on pain management within the CCU, to identify current barriers and facilitators for effective pain management. The students received a topic guide (see Supplementary Material) which was used to guide the interviews. The format of the interviews was semi-structured, meaning, the students could adjust their questions or add impromptu questions based on the role of the participant they were interviewing or to investigate topics the participant raised that they were unaware of. During the interviews, probes provided by the QI team were discussed (section 3.3). From the information gathered during the interviews, students proposed novel visualisations to support pain management.

The interviews took place in January and February 2020 at University College London Hospital, with each interview lasting on average 30 min.

### 3.3. Interview Probes

This research used probes that were produced by the QI team as part of a QI initiative within the CCU. These probes were designed to enable QI team member to review patients' pain levels and identify patients who either needed to have their pain re-assessed or were in severe pain, so that bedside nurses could be alerted and rectify the situation. Their probes were also used to identify trends within the CCU, allowing the QI team to investigate ways of improving pain management across the entire CCU. The data used to produce the probes was generated by the QI team from existing data, which only QI team members have access to. It was important to the QI team that the data from the CCU could be displayed on a weekly basis to provide an ongoing analysis of performance. Initially, Statistical Process Control charts, typically used in QI, were used, but found to be poorly understood by clinicians. To address this, they presented the data in different formats hoping they would be more intuitive to clinicians and would help with trying to unpick why specific patterns were being observed.

**Figure 2:**
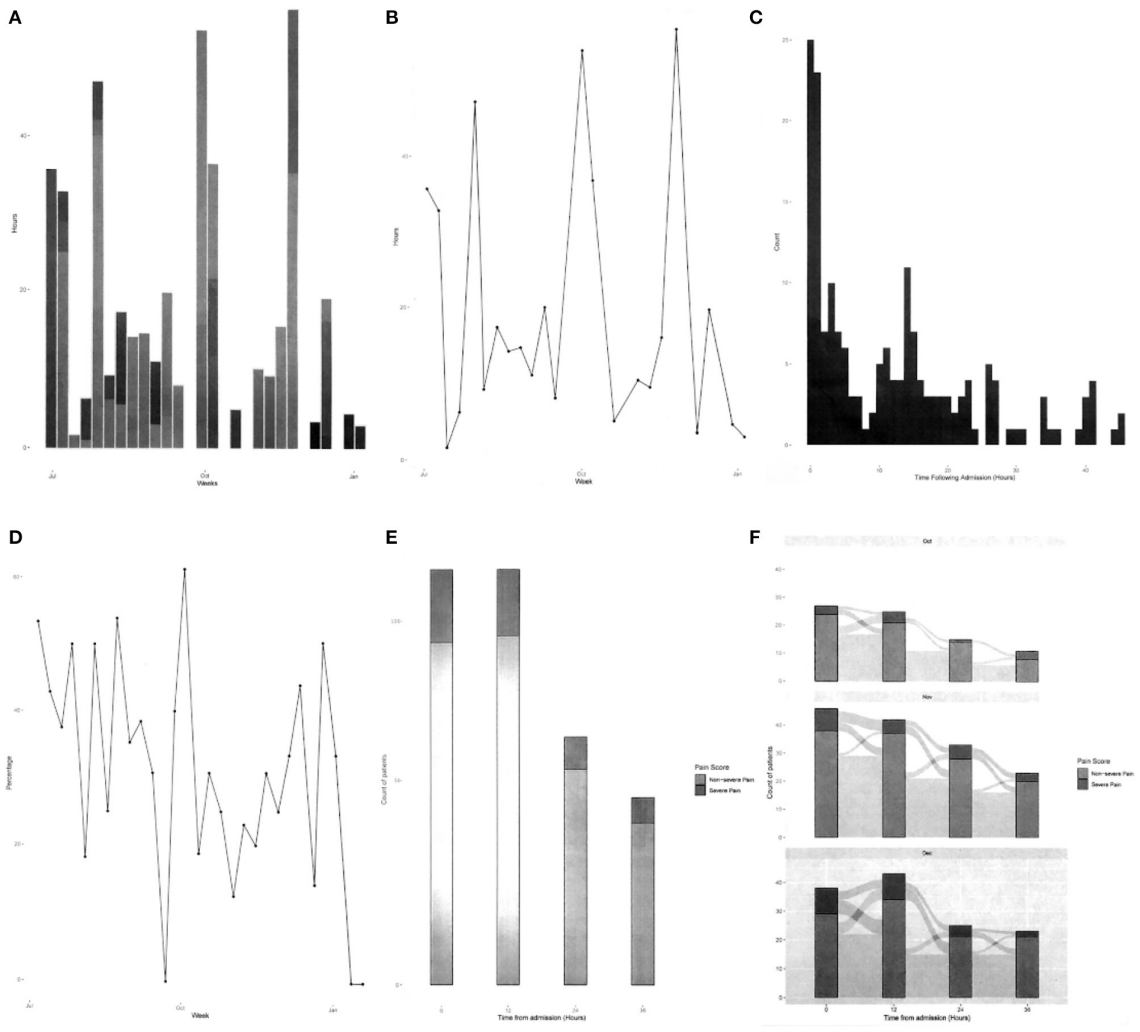
Greyscale graphs used during the interviews, **(A)** total hours of severe pain, **(B)** cumulative hours of pain, **(C)** time to severe pain after admission, **(D)** severe pain in 48 h period, **(E)** change in pain scores in first 48 h, and **(F)** patients' pain journey over 48 h period.

The newly formatted graphs were used as probes during the interviews, allowing participants to discuss what information was easy or difficult for them to understand, as well as identifying what important information was missing. The probes used in the interviews consisted of different styles of graphs to portray various aspects of patient pain within the CCU and were printed on A4 paper and presented in greyscale to participants as shown in Figure 2. The QI team's desired outcome was to develop a bespoke dashboard, displayed in the CCU, where staff can observe changes in real time and in response to various interventions.

### 3.4. Analysis

Two sets of analysis were conducted on the interview transcripts. The first was by the Masters students as part of their coursework, and the second was by ourselves, independent of the students' work. Once both analyses were complete, a triangulation of the findings was conducted to identify any issues overlooked in either analysis.

The students analysed the transcripts based on the COM-B behaviour change model (Michie et al., 2011). This involves identifying three components that shape behaviour: capability, opportunity, and motivation. The students analysed their transcripts in terms of these three components and determined which were barriers and enablers for effective pain management.

We conducted a thematic analysis of the students' transcribed data, prior to reviewing the students' outcomes. The purpose of the analysis was to identify users' needs and to understand what information they rely on to understand patients' pain levels, how the probes might help them elicit patient needs and CCU trends, and how they think dashboards could be improved to achieve these tasks. Each of the twelve transcribed interviews were analysed to gain insight, from which six broad themes were identified. Then, using NVivo, a qualitative data analysis software package, a coding scheme was developed that focused on six key areas: determining patient pain levels, both verbal and non-verbal; how pain is managed in the CCU, including issues with current processes, risks associated with pain management, and desired changes; patient issues with pain management; training issues; recording pain scores into the Electronic Health Record (EHR); and reviewing and interpretation of pain information. From this coding scheme, common themes of interest were collated and key issues from the participants' perspectives were highlighted. The focus for this paper is on issues relating specifically to the management, understanding, and analysis of pain in the CCU from a QI perspective.

Comparing the students' findings and our own, we found that each student group drew mainly on the information they gathered from their own interviews. This resulted in each group identifying different areas of improvement and developing those in depth. As our analysis was based on the full corpus of interviews, it bridged gaps between the students' work.

Triangulating across the different analyses enabled us to synthesise a design proposal that incorporates all factors raised in the interviews using known visualisation techniques, including building on the students' work. The visualisations support the exploration of patients' pain during their time in the CCU, focusing on QI.

The structure of the analysis follows the design study methodology laid out by Sedlmair et al. (2012), who prescribed a nine-stage framework made up of three top-level categories. This framework provides guidance to ensure that the problem we are trying to develop visualisations for is robust, so that we can avoid potential pitfalls. They define a design study as '*a project in which visualisation researchers analyse a specific real-world problem faced by domain experts, design a visualisation system that supports solving this problem, validate the design, and reflect about lessons learned in order to refine visualisation design guidelines.'* To assist in identifying the key requirements for each visualisation, a task typology developed by Brehmer and Munzner (2013) was also adopted. This typology clarifies the tasks the QI team wish to perform and identifies *why* they are performed, *how* they are performed, *what* is required to perform the task, and the expected result of that task.

## 4. Results

Our analysis of the data led us to identify three key tasks the QI team can perform in planning and monitoring quality improvement for pain management. These are:

1. Understanding patterns over comparable patients to identify specific areas of concern in practice (e.g., of managing particular kinds of situations or patient conditions) (section 4.1).
2. Understanding overall ward performance in terms of pain management to identify ward processes that need attention (e.g., frequency of pain scoring) (section 4.2).
3. Reviewing the quality of documentation of pain levels, specifically, existence of pain scores, timeliness of scoring, and accompaniment of supporting documentation. This data was also recognised as defining the integrity of the pain management data (section 4.3).

These three themes are covered in the following sections. We have drawn on the work of Brehmer and Munzner (2013) to structure the visualisation tasks using their typology. The typology for each task is produced with the nodes relating to *why* they are performed in yellow, *how* they are performed in green, and *what* the tasks inputs and outputs are in grey. It should be noted, that the identified tasks are independent of each other, meaning that a task does not need to be completed in order to accomplish the next task.

### 4.1. Identifying Patient Trends

QI team members wish to improve pain management for patients, but currently don't have the necessary information to make informed decisions. P7 voiced this by stating “As a nurse, I'm concerned about my patient. But I think, as a unit, we should be looking at everything.” This highlights that in order to improve patients' pain levels we need to incorporate as much relevant information as we can.

Participants highlighted five key elements that are used when identifying and managing patients' pain levels, which are: pain scores, pain duration, capabilities of the patients, the type of pain relief and dosage, and time staying in the CCU. Within the CCU, pain scoring ranges from 0 to 4, with 0 representing no pain, and 4 indicating severe pain; and bedside nurses regularly check a patient's ability to perform the three tasks of coughing, deep breathing, and regaining mobility. The results represent the progress in the patient's ability to meet the criteria required to be discharged from the CCU. These aforementioned elements are currently used by the pain team to make decisions, with P7 stating “The pain team will look at the amount of pain relief that the patient has had and the pain scores, and would make decisions based on that.” Adding to this, P10 shared that they also look at “the level of pain patients have been in; the length of time they've been in [the CCU]; and where [they are] in their [CCU] journey.” All of this information is currently captured and inputted into the EHR. However, the QI team does not currently have a system that allows them to inspect this data and compare patients' CCU journeys to identify specific areas of concern. Additional factors, although not mentioned by participants, that are both available on the EHR and relevant to pain relief could also be included: the surgery the patient received, the clinician who performed the surgery, and patient specific attributes, such as age, sex, and weight.

Having the ability to inspect and contrast patients' pain journeys while in the CCU has the potential to identify currently unidentified causes of pain for patients. P5 pointed out that with the right tools it “could inform how we change our practices as a whole.” P7 remarked that through the QI team analysing data of both current and previous patients “we will be able to kind of predict how certain types of patients are gonna behave.”

**Figure 3:**
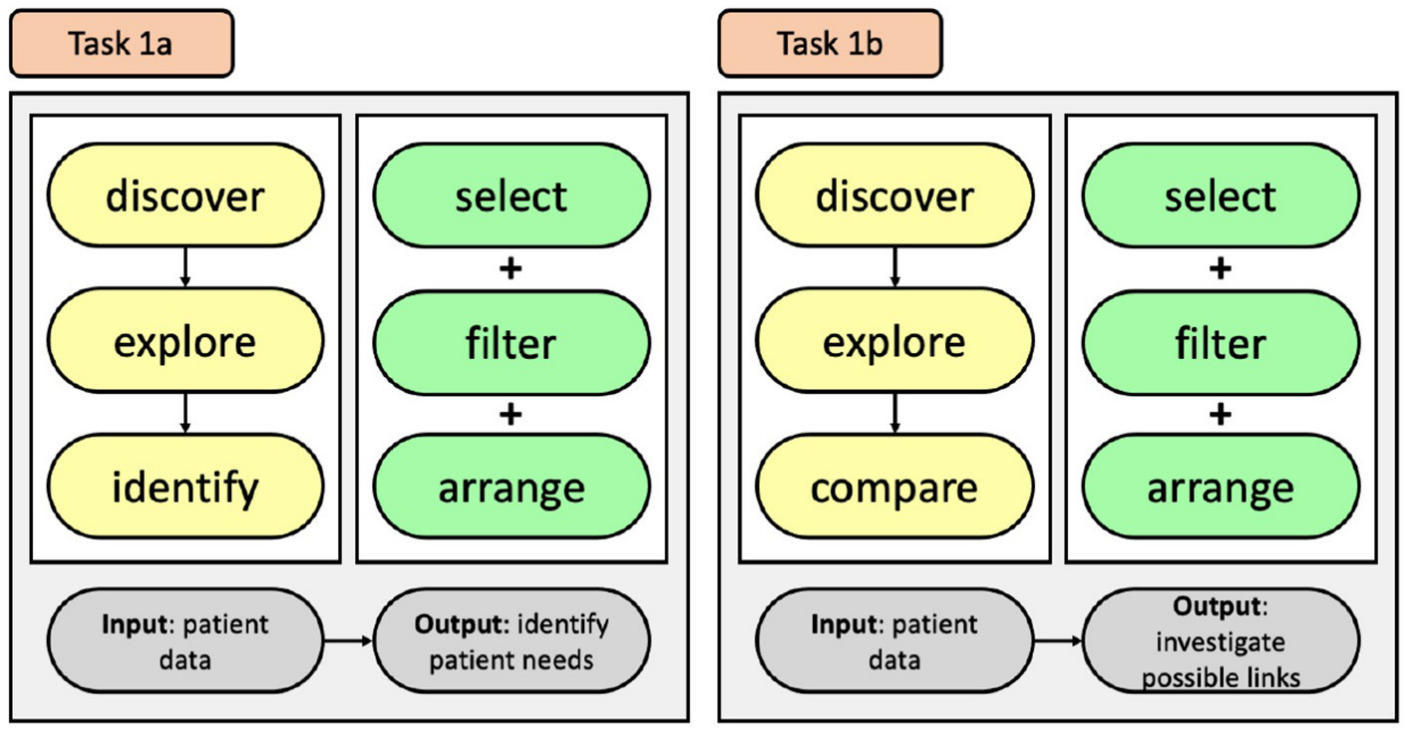
Tasks used to identify patient trends.

Based on the interview data, it was clear that the QI team believe that by comparing patients' information, trends relating to pain management can be uncovered. However, there are also times the QI team wished to inspect individuals. This information highlights two tasks the QI team want to achieve and identifies why they would use an information visualisation, how they will achieve the tasks, and what we expect will be a result of both tasks. The QI team want to discover trends relating to pain management that they can use to inform changes to their current processes. This is accomplished by exploring the data and then comparing results from patients to inform decisions (Task 1a); also, when inspecting individuals, the exploration may lead to identifying individual patient needs (Task 1b), see Figure 3. They can achieve these by selecting, filtering, and arranging data that is available on the EHR.

### 4.2. Identifying Ward Trends

To allow the QI team to make informed decisions on how to improve pain management within the CCU the data they are investigating needs to be clear to minimise the risk of misinterpretation. The probes generated for this study focused on ward trends, and were developed by the QI team to highlight the pain management data they felt was important. However, participants found the probes difficult to comprehend, with P3 stating “The average person doesn't have a clue how to interpret it. I sit in management meetings every month and look at heat maps and all of these different things, if you have someone there explaining it to you, you go oh yeah, lightbulb moment.” P11 noted that “Five different people with reasonable understanding of data could come up with different interpretations of what they see.” Therefore, to improve sensemaking and remove ambiguity of results, visualisations of the data need to be improved.

There were key issues that made it difficult for participants to decipher the meaning of the probes, with most participants unable to identify what the graphs were detailing. A number of participants provided insights into the difficulties, with P10 explaining “It doesn't state how many patients are on the left-hand side. or there's no scale to the left-hand side so. you can't judge. there's no context on the side.” P5 expressed their thoughts stating, “the numbers are too small yeah. Letters are too small. It's all wrong.” Finally, P7 gave their opinion saying “The charts look really busy and they have a lot of scores that we've never used in this [CCU].”

From these perceptions it was evident that visualisations need to include key supporting details so that users can frame the results in the correct context. Also, supporting details such as titles and captions need to be appropriately sized. Finally, the parameters of the metrics need to support the user's knowledge of pain management and not include unfamiliar terminology that can inhibit sensemaking.

Participants provided some suggestions they felt could help improve interpretation of the probes. Both P1 and P2 suggested the addition of timescales with P1 stating “If it was broken down into shorter time periods, then that would be easier.” With P2 supporting this with “It would be nice if we could say how many hours this bit is, probably by actual week or actual months.” P5 also provided a suggestion stating, “we need to find better and smarter ways of displaying the data in a way that you can interpret it usefully. a little bit clearer. and I think visually attractive too. making it really clear and really simple is the right way for not making it too clever, not making it too complex.” Adding in the suggested features, along with the filtering features suggest in section 4.1, would allow the users to explore the data in a more meaningful way, and provide them with more control of the data they are examining.

By incorporating these suggestions, we have the opportunity to provide the QI team with tools to support sensemaking, thus enabling the CCU to improve in ways they currently are unaware of. P5 shared an example of how exploring data allows the QI team to see what is actually happening, as paraphrased in the following sentences. The hospital is interested in a particular antibiotic called meropenem. It is a powerful antibiotic, but they don't want to overuse it. The doctors think that they don't prescribe it often, but upon reviewing the data they realise that, overall, they have prescribed this antibiotic more than anything else, so their impressions were completely wrong. P5 then related this to pain management, “I think we treat pain very well. But the data might well tell me something very different. That's very dangerous. We've never had data that allows us to examine what we are doing. We're very subjective. We need objective evidence.”

By focusing on these key concerns, the QI team would be better equipped to conceptualise the data to support their sensemaking, which in turn would result in more robust decision making to improve pain management within the CCU.

**Figure 4:**
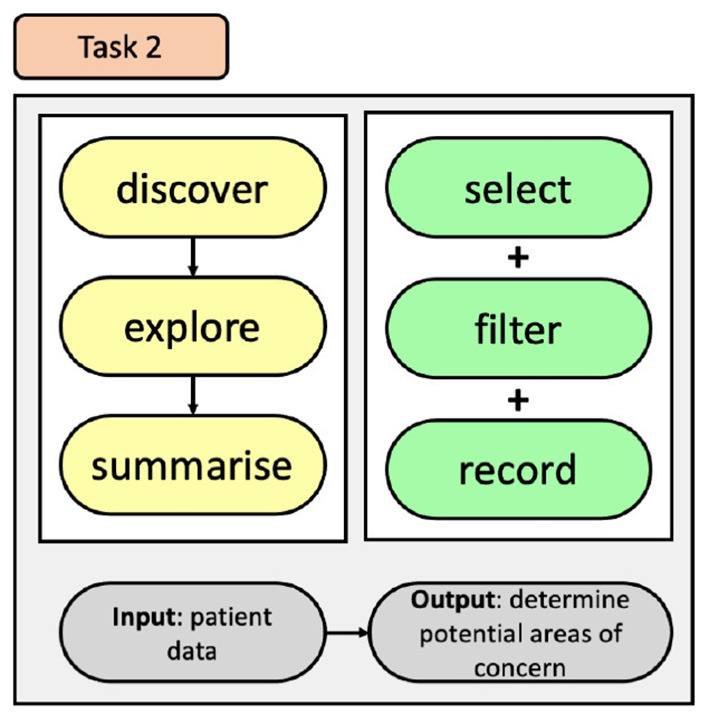
Tasks used for the exploration of pain data within the CCU.

To improve the users' ability to recognise trends and areas of concern within the CCU, relating to pain management, we have developed a task that incorporates the identified user needs to permit them to better explore CCU EHR data, see Figure 4. This task offers users the ability to discover information through exploration of CCU data by incorporating modes of selecting and filtering the data, and then affording them the option of recording their search queries and results. Doing so will provide users with control of their exploration and help them understand the impact of their choices.

### 4.3. Requirements for Documentation

Obtaining accurate and timely pain scores is necessary to ensure high quality pain management within the CCU. At the hospital where this study was conducted, pain scores are gathered every 4 h by bedside nurses or within 30 min of pain relief administration to ensure that the pain relief was working satisfactorily. The scores are then inputted into the EHR. However, the current process does not seem effective. P9 remarked that they believe that “a lot of people forget” to check pain levels; P2 had a similar opinion, saying “most of the staff in the CCU are not actually doing pain score as they should.” P3 raised a similar concern by stating “pain scores are one of the things that we found difficult to get anyone to record.” Until relatively recently, pain management data has been captured on paper, and the transition from paper to digital has made it harder for them to record patients' pain levels. P2 supports this assumption stating “not all of the nurses are very good with [recording data in the EHR], I think that's one of the problems we are having now.”

Although participants did not directly ask for a visualisation to help identify pain scoring issues, it was highlighted as a major concern relating to pain management, especially with the introduction of the EHR system. Providing the QI team with means to identify how well they are capturing pain scores has the potential to significantly improve pain management by highlighting areas where additional training may be required.

**Figure 5:**
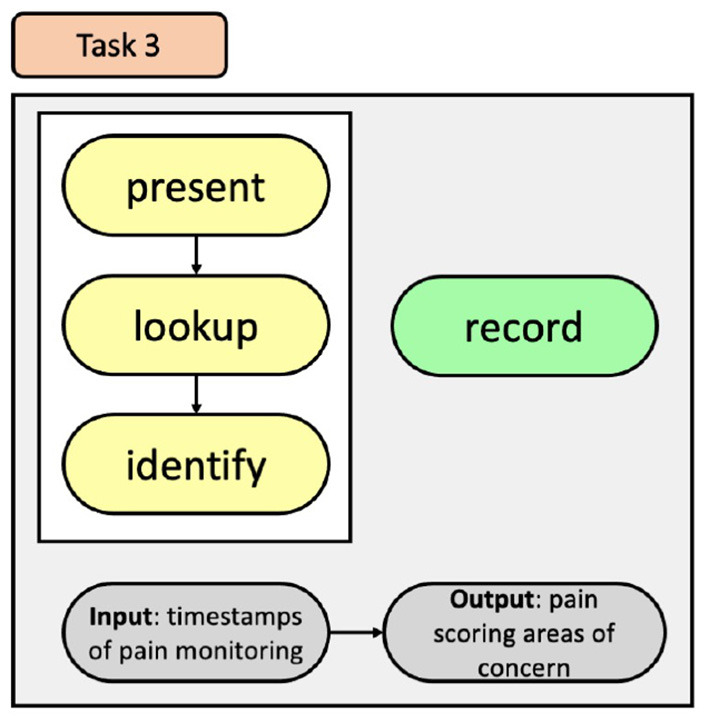
Task used to identify areas on pain scoring that need attention.

To indicate to the QI team how accurately data is being captured by the bedside staff, we developed a task, see Figure 5, that will present details relating to the timing of pain scores to allow for easy lookup and identification of the CCU's progress and allow the QI team to identify areas of pain scoring that can be addressed.

It became apparent that the healthcare professionals believed that with better access to exploring data they could gain better insights into the current practice of pain management. Therefore, to identify trends, additional requirements are: provide the ability to compare and contrast patients' data, for both current and discharged patients; include additional information relating to patients to provide more context; visualisations should be clear with appropriate sized headings; and data needs to be captured effectively and regularly.

## 5. Dashboard Proposals

Based on the information provided in the results, we developed dashboard proposals to help navigate pain data. These should, in turn, enable the QI team to understand core issues, make informed decisions, and generate actions they can test and evaluate. These dashboards are providing one of the core qualities expressed by Randell et al. (2020) by bringing data from the ward to the board, where we are considering the QI team to be the “board” as they are making decisions based on the information they uncover.

The analysis highlighted that there are three areas in which dashboards can be used to improve the QI team's sensemaking of pain management within the CCU. The first dashboard allows the QI team to compare and contrast individual patients to identify patient trends. A ward overview provides the QI team with the ability to explore how pain management can be improved within the CCU over a long period of time and could also enable the team to identify the effects of any changes they implement. The documentation dashboard investigates the performance of staff reporting pain scores and could be used to ensure the integrity of the pain data.

### 5.1. QI CCU Patient Overview Dashboard

**Figure 6:**
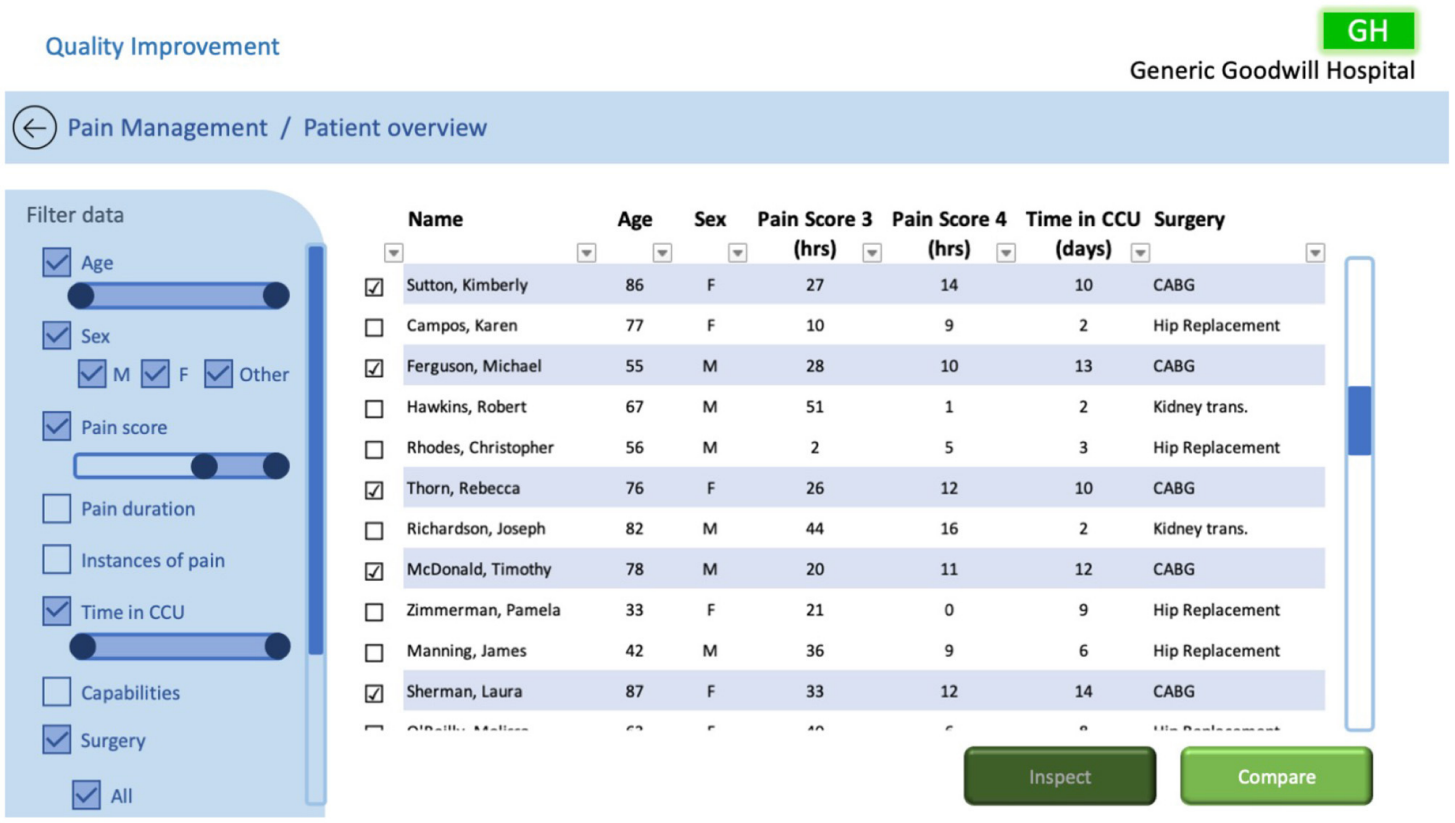
QI CCU patient overview dashboard.

Tasks designed to compare and contrast individual patients to identify trends were established in section 4.1. To achieve the defined tasks, we propose a dashboard, such as that illustrated in Figure 6, that empowers the QI team to inspect individual patients, both past and present, and compare selected patients. Doing so should enable the QI team to investigate possible links relating to patients' pain journeys within the CCU.

As described in section 4.1, the dashboard was required to facilitate discovery via exploration and comparison of data. To achieve this, selecting and filtering of EHR data was included so that the QI team could identify trends and compare possible links between patients, relating to pain management, to inform changes to their current processes. The dashboard includes the data the pain team currently use to treat patients' pain levels, but also includes other relevant data from the EHR. With this information the QI team can select the data they want to investigate, filter the data based on their requirements, and arrange the data by sorting the specified fields (either ascending or descending). The dashboard also includes design requirements specified by Randell et al. (2020), specifically: choosing performance indicators, assessing performance, identifying causes, and communicating from ward to board.

In this example we have filtered the patients by age, sex, pain score, their time in the CCU, and the type of surgery they received. The pain score is filtered to only show patients who had pain scores of 3 and 4, showing the accumulative number of hours for each of the patients' pain scores during their time in the CCU. The filtered information is tabulated so the user can inspect the data and add additional filters if required. Using the checkboxes, the user can either select an individual patient and review their individual data with the inspect function or select multiple patients they wish to compare further by selecting the compare function.

**Figure 7:**
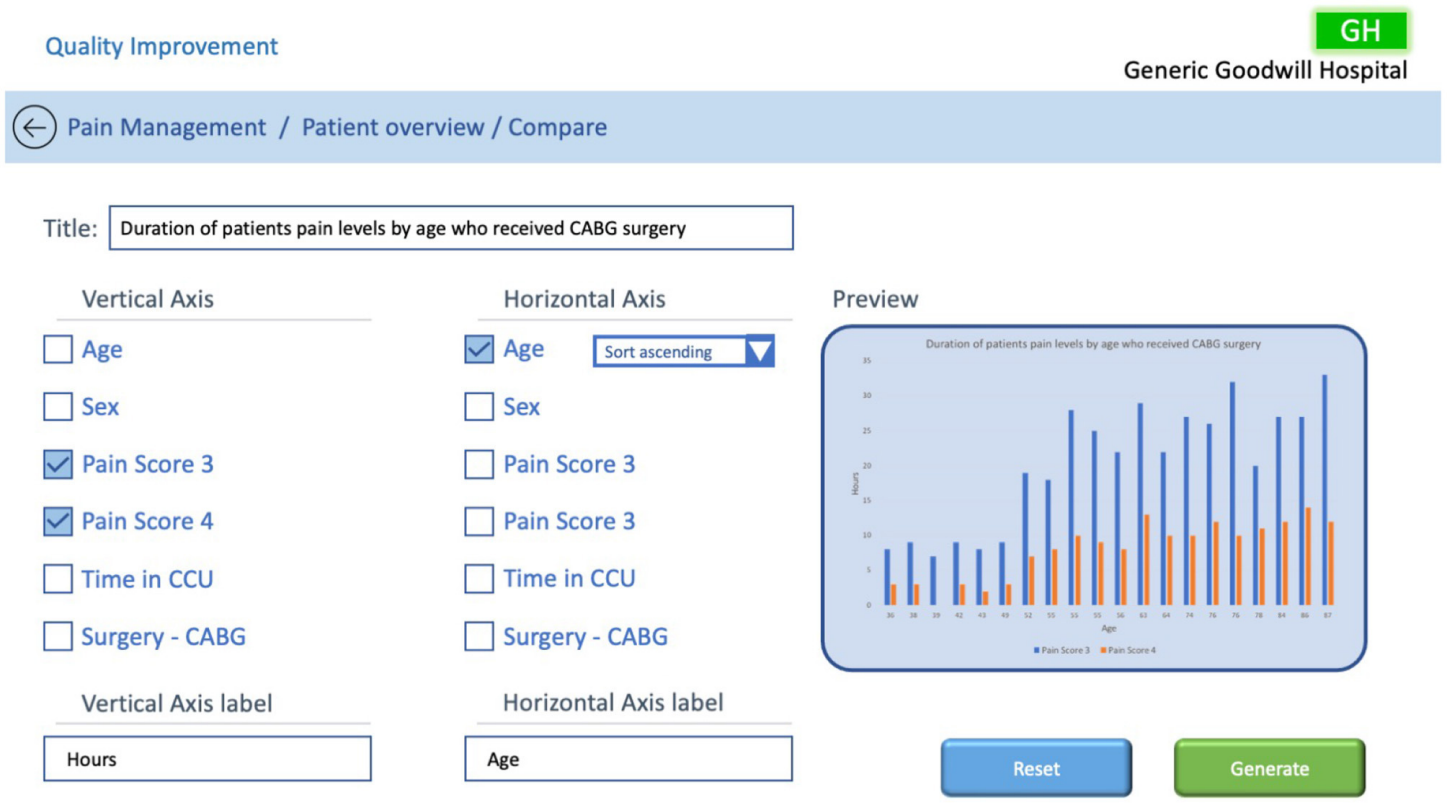
QI patient comparison dialogue screen.

The compare function would open a new dashboard and allow the user to drill down into the data to try and identify trends. In the example in Figure 7, the user has identified from the previous screen that patients who received coronary artery bypass grafting (CABG) surgeries had a high number of hours in high pain and wants to explore further to try and discover the root cause related to CABG surgery. To assist them in identifying issues they have selected to compare patients by age. The compare dashboard may allow the user to select which of the metrics they wish to compare, based on the previous filters, and add titles and labels to the output to provide meaning to the comparison. To improve sensemaking, a preview of the output would provide a predictive and perspective visualisation. Once the user is satisfied with their decision, they can generate the graph using the selected patients' data.

**Figure 8:**
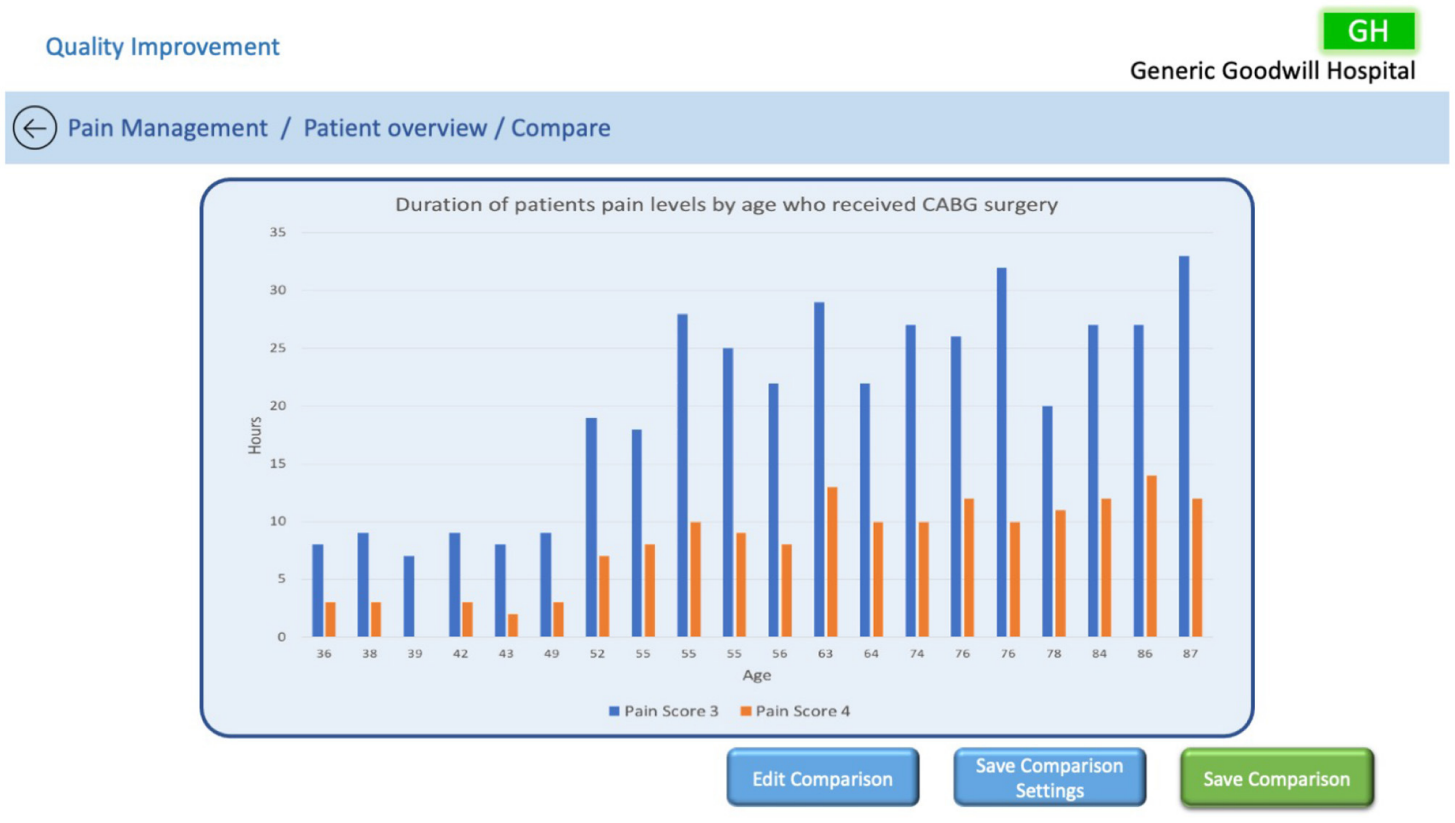
Example of QI patient comparison output.

A dashboard could enable the QI team to explore patient data to identify trends that they otherwise would not be able to discover. For example, the QI team might filter the users by age, a particular surgery, and pain scores to find that patients between the ages of 35 and 50 appear to have better controlled pain management than those older after receiving the same surgery. A depiction of this scenario can be seen in Figure 8, where the user has the ability to edit the comparison, save the comparison settings so they can be applied to other patients, or save the comparison with all of its associated data. This type of information can then be communicated from the ward to the board to make a change to current processes—for example, to change the dosage of medication prescribed post-surgery for older patients to better manage their pain levels.

### 5.2. QI CCU Ward Overview Dashboard Examples

In section 4.2, we developed a task that would allow the QI team to explore CCU data to identify weaknesses, opportunities, and trends relating to pain management. To achieve this task, we have proposed a number of dashboards based on the probes to improve the users' comprehension of the information they were‘exploring.

The design of the dashboard was influenced by the users' requirements, as described in section 4.2, and takes into consideration design requirements presented by Randell et al., specifically: evaluating trends over time; an adjustable time period; filterable data; and the ability to drill down into data.

**Figure 9:**
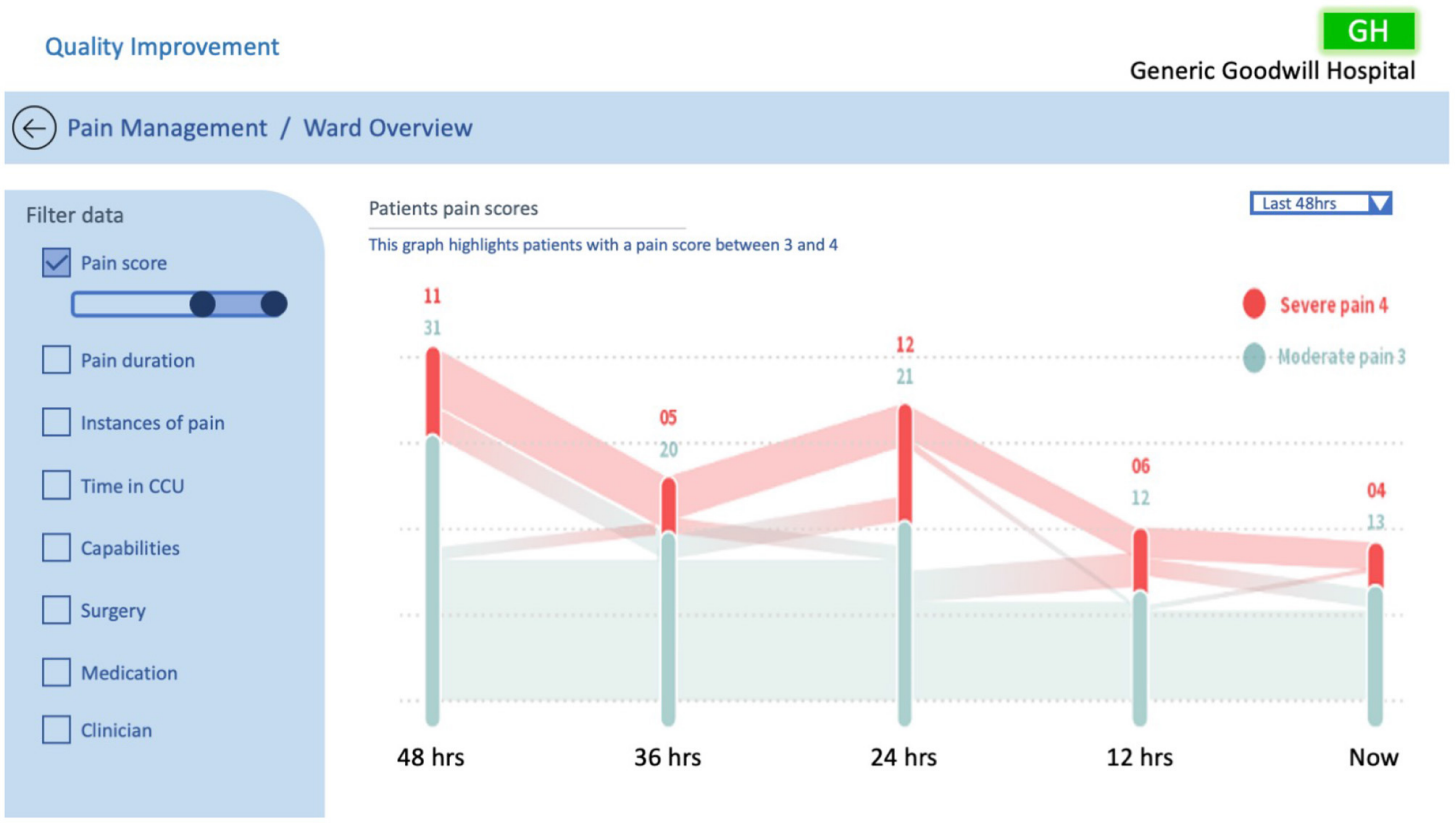
QI CCU ward overview example #1, investigating patients pain scores.

**Figure 10:**
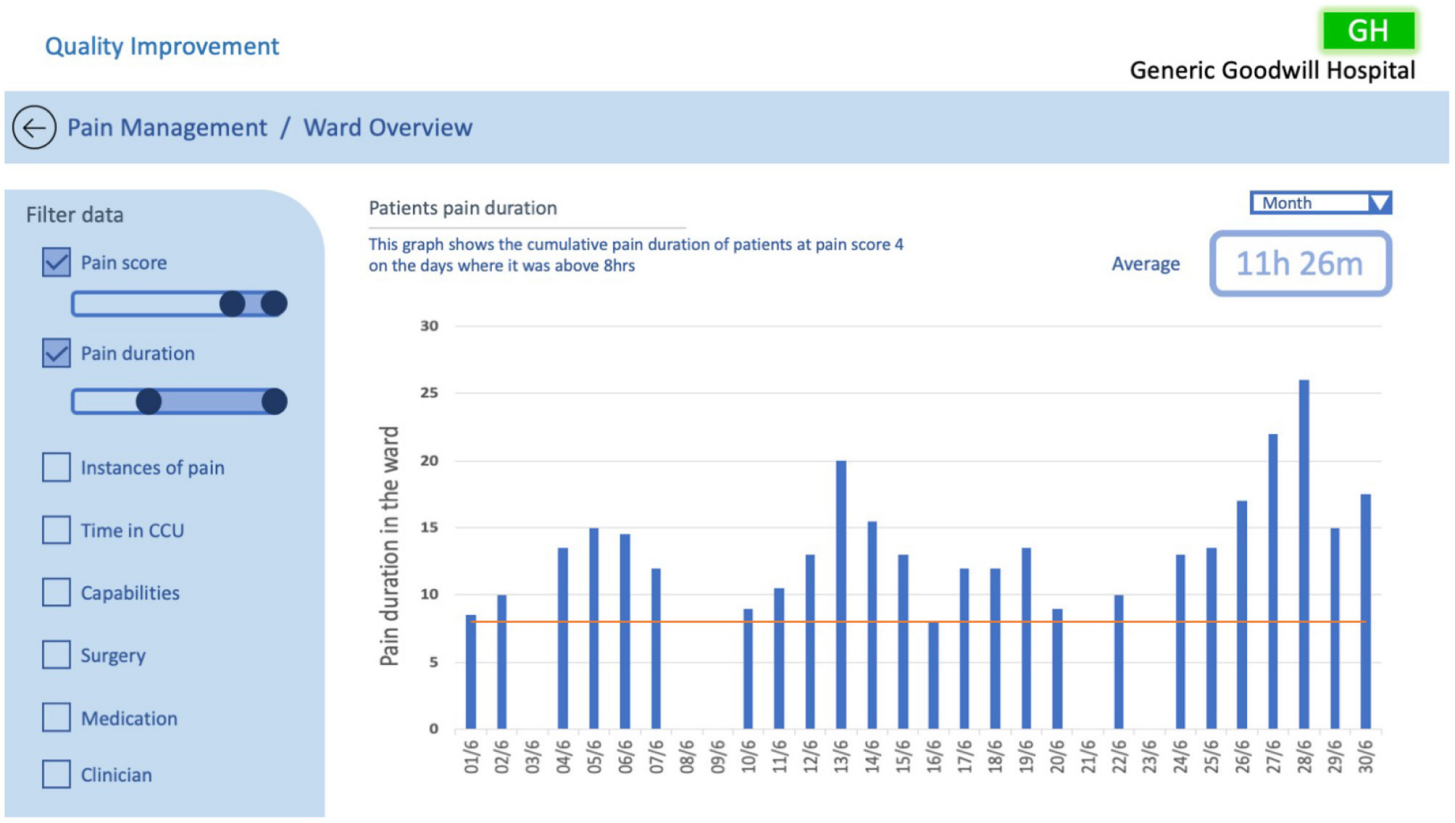
QI CCU ward overview example #2, investigating patients pain duration.

**Figure 11:**
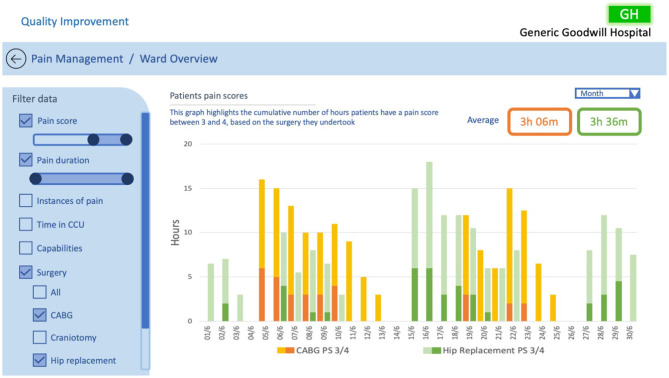
QI CCU ward overview example #3, investigating patients pain scores based on surgical treatments.

**Figure 12:**
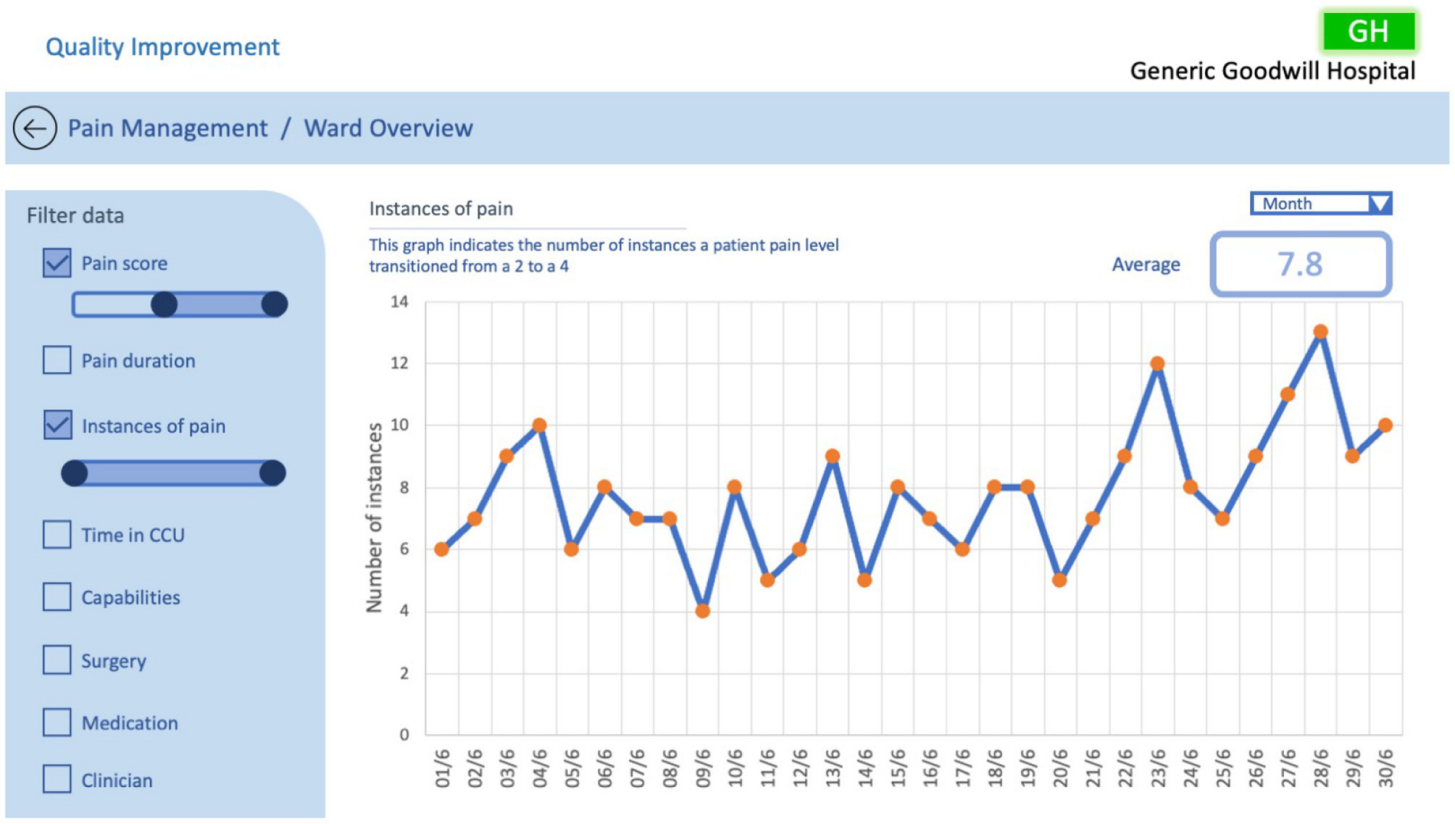
QI CCU ward overview example #4, investigating instances of high pain score transitions.

In the interviews, participants highlighted that their ability to quickly assimilate and understand information presented in the probes made it difficult to come to any conclusions regarding pain management and expected that everyone would interpret the data differently. The probes included unfamiliar information, were aesthetically unpleasing, complicated, and provided no clear context. In Figures 9–12, we illustrate a variety of examples that build on the data representations included in the probes, focusing on data considered favourable for the QI team, to overcome participants' concerns. The examples make use of adjustable time scales that allow users to inspect data from both current and past patients. Filters based on terms familiar to the QI team are utilised including: pain score, pain duration, the clinician, the surgery, instances of pain transition, medication, and patient capabilities. The filters would allow the QI team to investigate data from different perspectives, to gain more informed insight into current pain management procedures. Drilling down into the filtered data output is a desired function, which would enable the QI team to inspect the data with more detail. The examples are designed to allow the QI team to better conceptualise the information by providing clear understanding on what they are visualising to support sensemaking and embolden them to explore pain management data to determine weaknesses to resolve and opportunities for improvement.

Figure 9 represents the probe in Figure 2F in visual form illustrating the pain score journey of patients in the ward with a pain score of 3–4 over a 48 h time period. A Sankey graph has been utilised as it allows the user to both visualise the values and track the movement of patients' pain scores over time. This takes into consideration the design principles of filterability, adjustable time period, timely data, the ability to support clinical discussion, and analysis of trends over time, as outlined by Randell et al. This style of graph was chosen as the healthcare professionals reported finding it easy to interpret and it allowed them to quickly visualise the pain history of the entire ward. Likewise, this example is beneficial for the QI team for their performance analysis, specifically to quickly and effectively identify spikes of pain in the CCU over the last 48 h.

The examples in Figures 10, 11 illustrate two scenarios of cumulative hours of pain in the ward as denoted in the probe in Figure 2B. Figure 10 presents patients who had a pain score of 4 (severe), only showing days when the cumulative pain duration in the ward exceeded 8 h. Similarly, Figure 11 displays patients with a pain score of 3 and 4, but with the addition of surgery comparison. In this example we have illustrated the selection of CABG and hip replacement for comparison; however, any combination of surgeries could be used. These two examples utilise the same design principles specified in Randell et al. as in the previous example, with the addition of the ability to drill down into data and compare surgical procedures. This design was chosen as the healthcare professionals wanted to understand how well the ward was doing at controlling the pain levels of patients. Furthermore, the example in Figure 11 enables users to discover trends relating to particular surgeries and determine whether the pain management for those surgeries was adequate. This would enable the QI team to uncover trends specific to surgeries that may be hidden when viewing general pain duration data; this could be utilised to identify opportunities for improvements in the approaches taken to specific surgical pain relief management. This information could also help to understand the nature of spikes in high pain scores and promote further investigation.

Figure 12 depicts the number of instances in a ward where a patient's pain scale has increased from 2 to 4 over the selected period, as represented in the probe in Figure 2E. A line graph is utilised to illustrate the frequency in which patients' pain levels are transitioning from a manageable level to a severe level allowing the QI team to easily identify trends and patterns in the ward pain management, with orange circle markers indicating points that allow for further investigation. This example implements Randell et al.'s design principles of filterability, adjustable time period, the ability to drill down, timely data, support clinical discussion, and analysis of trends over time to support sensemaking and conceptualisation. The information in the example enables the QI team to focus on the patients where spikes in pain elevation occurs, potentially helping them understand what factors are contributing and hence develop solutions.

There are two key points that need to be addressed when changes to pain management are implemented: (1) saving and recalling of dashboard settings, and (2) each implementation must be well documented. This would allow the QI team to understand the impact of the adjustments, and correlate actions and effects. The ability to recall existing data and run the same filters over current data is essential. Having saved filters can also provide deeper insights into data, allowing long term issues to become more visible. Therefore, these two points are additional requirements; although not found during the interviews, they play a pivotal role in understanding the effectiveness of process modifications.

### 5.3. QI Documentation Dashboard

**Figure 13:**
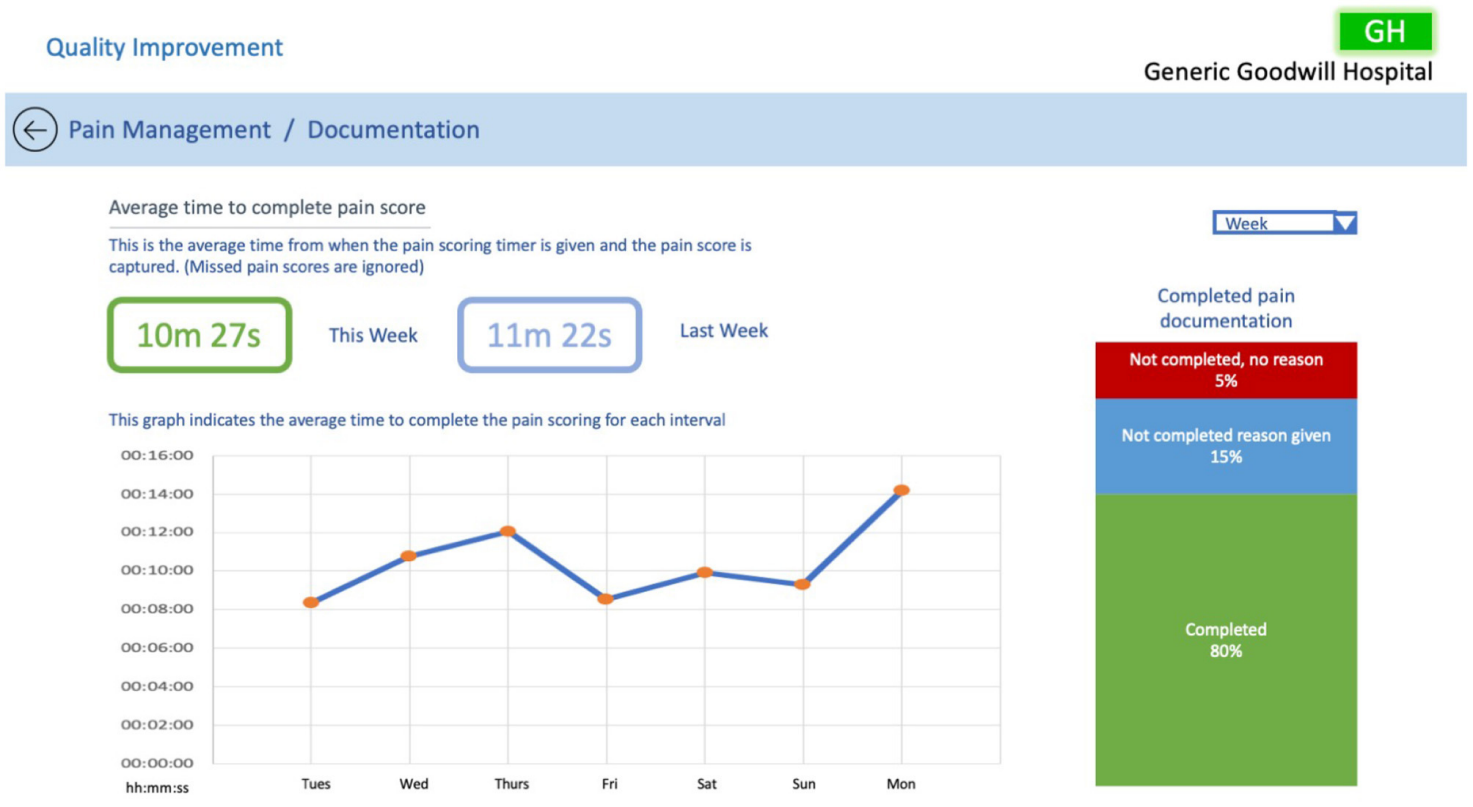
QI documentation dashboard.

Determining how effectively bedside staff are entering pain scores was identified as a task that could help the QI team improve pain management within the CCU. To accomplish this task, we have proposed a dashboard, see Figure 13.

The dashboard presents the average time it takes bedside staff to enter pain scores, and allows the QI team to evaluate timings over the set time period. Additionally, the graph allows the user to select points of interest to drill down further into the data. For example, clicking on a date of interest can present all of the bedside nurses on shift that day and share their individual scoring times. This may indicate that a bedside staff member may be struggling to complete their pain scoring, and allow the QI team to arrange assistance to that staff member. The dashboard also indicates whether supporting documentation has been entered along with pain scores.

Aside from highlighting bedside staff issues, this dashboard can also be used to determine the integrity of the pain data. This information is important to the QI team as it provides them with confidence in the quality of the pain management data they are investigating. This ensures that any changes the team implement to processes within the CCU based on the pain management data are effective. For example, if during exploration of pain management data the QI team notice spikes in patients' pain levels, they can evaluate the timeliness of pain scoring over that period. If during this period pain scores have been captured on time it would indicate that the integrity of the data is probably high, providing confidence that any changes made to processes can have an impact on improving pain management.

## 6. Discussion

The probes used during the interviews highlighted the concerns of healthcare professionals in trying to understand pain management within the CCU. The results indicated that the main areas of weakness from the participants' perspectives were that the probes included unfamiliar information, were aesthetically unpleasing, complicated, and provided no clear context. This led to confusion and misunderstanding of the data. During discussion with the participants on their desired outcomes, it became clear that they wished to gain more insight into the data through comparisons and contrasts. To achieve this, we adopted the design requirements developed by Randell et al. (2020) for QI dashboards.

The dashboard proposals take into account these design requirements which included: choosing performance indicators, assessing performance, identifying causes, communicating ward to board, and data quality. The examples shown in section 5 address these specific requirements. The patient and ward examples allow the users to choose their performance indicators by filtering the data in a meaningful way based on their inquiry. They allow users to assess performance by providing data over time, with a means to adjust the time period, and adjust the filter settings for specific evaluations. Root causes can be identified by drilling down into the data, enabling the QI team to interact with all the data on the EHR system. The dashboards promote the communication of data from the ward to the board, considering the QI team as the “board” as they are making decisions based on the information uncovered. Data quality and timeliness of the data are essential requirements to ensure that the QI team is working with information they can trust. As all data is available on the EHR, meaning that the moment data is entered at the patient's bedside it can be accessed by the QI team, data can be delivered in a timely manner. With regards to data quality, the dashboard proposal in section 5.3 helps to assess the integrity of the data to ensure it is quality data, which is crucial for the QI team as they need to ensure they have confidence in the pain management data they are investigating. The purpose of introducing these design elements is to improve conceptualisation of data for the QI team, who will utilise it in their decision making processes to improve pain management in the CCU.

Although Randell et al.'s design requirements do not include aesthetics, participants highlighted the need for clear visualisation of data to support their sensemaking. This was incorporated into our proposed dashboards through the selection of graphs which are familiar and straightforward, and through the use of coherent labelling, which provides context and enables the selection of familiar data.

The main focus of these dashboards is to promote sensemaking, as specified by Blandford et al. (2014). This is achieved by grouping the data into four modules that allow for better conceptualisation of information and focus on the priority outputs for the QI team.

Although pain scoring occurs at the bedside, the data that the QI team is interested in requires them to abstract away from the bedside to review trends and patterns. QI review can trigger behaviour change in the ward in terms of how pain management is perceived and undertaken in the future. Although such changes can provide better global advantages, they may unintentionally impact individual patients; evaluation of any changes need to be understood at both the ward level and the bedside level.

During the course of this research there have been practical challenges that made it difficult to examine the full potential of developing a QI-based dashboard for pain management. COVID-19 has had a major impact on the ability to conduct user-centred research, especially with healthcare professionals. The CCU involved in this study has undergone major changes in adjusting to catering for COVID-19 patients, which has diverted CCU staff from this project. Access to the technicians that is required to create these changes has not been possible, so we have been unable to work with them to integrate these dashboards directly with their current electronic health record (EHR) system and conduct the further evaluations that are needed. Despite these challenges, we have been able to present this work back to QI team members via Zoom conferences; from these, we have received positive responses and preliminary feedback on how these dashboards could provide improved conceptualisation in future iterations. Most of the feedback from the clinical team was positive, with QI team members expressing that they found the idea of splitting the data into defined areas a helpful perspective. However, some clinicians conveyed that this information was not useful to them in their practice, stating that rather than reporting on pain the focus should be on a patient's ability to perform the acts of coughing, deep breathing, and regaining mobility: as stated earlier, these results represent the progress in the patient's ability to meet the criteria required to be discharged from the CCU. It is beyond the scope of this paper to debate the different priorities of clinical staff in the CCU. Given the focus on pain management (a focus determined by the QI team), for future iterations we will work closely with the QI team in developing the dashboards to ensure that we have correctly identified and understood their problems and the tasks they are required to perform.

This study has identified key user needs for QI in pain management in intensive care and presented a design rationale and example instantiations of data visualisations that draw on both our own empirical evidence and guidelines proposed by others, notably Randell et al. (2020) and Blandford et al. (2014).

**Figure 14:**
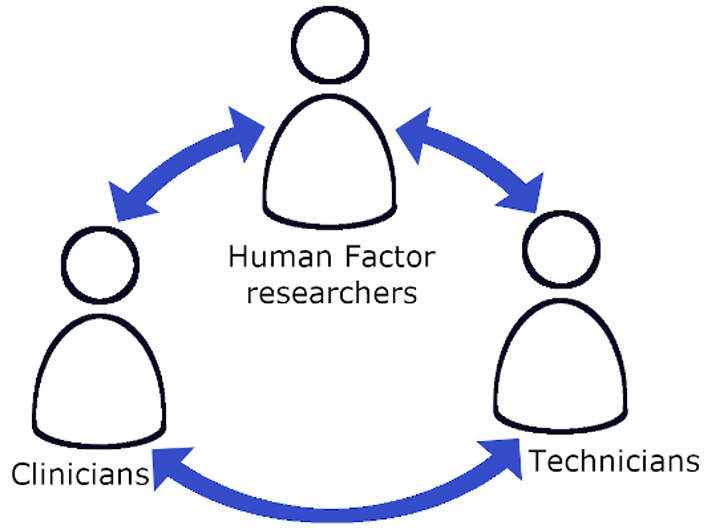
The interactions of a multi-disciplinary team required to develop new dashboards for the QI team.

To provide the best possible dashboards for QI teams to improve pain management, a multi-disciplinary team of clinicians, human factor researchers, and technicians is required as illustrated in Figure 14. Within this cluster, the clinicians provide their expertise in pain management and what data it is possible to collect, the human factor researchers can propose visualisations that facilitate engagement with the data in a meaningful way to support sensemaking, and technicians can incorporate these visualisations within the framework of the EHR. Inevitably, the data gathered so far has focused on participants' perceptions of their needs, rather than their validated needs. Just as the original probes (Figure 2) enabled participants to give their reactions to those representations, so, the visualisations presented in this paper could serve as a probe in a further round of evaluation. However, we believe that—once our clinical colleagues have capacity to invest further time in this project—it will be most beneficial to conduct future studies with high ecological validity (van Berkel et al., 2020). To achieve this, it will be necessary to make the dashboard available to QI teams as part of their routine work, populated with live patient data. This will require us to work closely with the EHR technical team to refine these prototypes in line with what is technically possible and integrate them with the EHR. It will then be possible to follow an iterative process of refining the dashboards to support sensemaking for the QI team within the constraints of an EHR system in a way that has high ecological validity.

## 7. Conclusion

This research has investigated the needs of the quality improvement team members to better conceptualise data to support sensemaking. This was achieved through analysing transcripts of interviews with CCU healthcare professionals. From the analysis we identified that the healthcare professionals found the probes, in their current format, difficult to interpret and they desire data which allows them to explore through filtering and data drilling techniques. We established that staff members believe more effective pain management strategies can be identified when data is presented in a format that improves sensemaking. With this understanding, we have developed QI-specific pain management dashboard proposals. These dashboards would allow the QI team to observe patient-orientated, longitudinal ward, and staff performance issues through navigation and exploration to make sense of the data that in turn could provide support for decision making.

As noted above, future work entails iterative testing of the dashboards with a multi-disciplinary group of clinicians, human factor researchers, and technicians to provide better visualisations integrated with the EHR to allow for richer engagement with the data to support sensemaking.

## Data Availability Statement

The raw data supporting the conclusions of this article will be made available by the authors, without undue reservation.

## Ethics Statement

The studies involving human participants were reviewed and approved by UCLIC Departmental Ethics Committee. The patients/participants provided their written informed consent to participate in this study.

## Author Contributions

AB, JW, and MB contributed to the conception and design of the study with support from MS and PV, who developed the probes used in the study. JO conducted the data analysis and wrote the first draft of the manuscript. All authors reviewed and contributed to manuscript revision and approved the submitted version.

## Conflict of Interest

The authors declare that the research was conducted in the absence of any commercial or financial relationships that could be construed as a potential conflict of interest.
